# Parallel *in vivo* monitoring of pH in gill capillaries and muscles of fishes using microencapsulated biomarkers

**DOI:** 10.1242/bio.024380

**Published:** 2017-05-15

**Authors:** Ekaterina Borvinskaya, Anton Gurkov, Ekaterina Shchapova, Boris Baduev, Zhanna Shatilina, Anton Sadovoy, Igor Meglinski, Maxim Timofeyev

**Affiliations:** 1 Institute of Biology at Irkutsk State University, Irkutsk 664003, Russia; 2 Institute of Biology at Karelian Research Centre of Russian Academy of Sciences, Petrozavodsk 185035, Russia; 3 Baikal Research Centre, Irkutsk 664003, Russia; 4 Institute of Materials Research and Engineering, A*STAR, 138634, Singapore; 5 University of Oulu, Optoelectronics and Measurement Techniques Laboratory, Oulu 90570, Finland

**Keywords:** Blood pH, Encapsulated fluorescent sensors, Interstitial pH, Microencapsulated biomarkers, Physiological measurements *in vivo*, Zebrafish

## Abstract

Tracking physiological parameters in different organs within the same organism simultaneously and in real time can provide an outstanding representation of the organism's physiological status. The state-of-the-art technique of using encapsulated fluorescent molecular probes (microencapsulated biomarkers) is a unique tool that can serve as a platform for the development of new methods to obtain *in vivo* physiological measurements and is applicable to a broad range of organisms. Here, we describe a novel technique to monitor the pH of blood inside the gill capillaries and interstitial fluid of muscles by using microencapsulated biomarkers in a zebrafish model. The functionality of the proposed technique is shown by the identification of acidification under anesthesia-induced coma and after death. The pH in muscles reacts to hypoxia faster than that in the gill bloodstream, which makes both parameters applicable as markers of either local or bodily reactions.

## Abstract (precis)

**Summary:** An optical technique for the simultaneous *in vivo* monitoring of pH in the blood of gill capillaries and interstitial fluid of muscles developed and tested in zebrafish.

## INTRODUCTION

Implantable optical nano- and microsensors are novel tools and provide the possibility for obtaining repeated measurements of various physiological parameters for certain tissues in a broad range of species (Gurkov et al., 2016; Ruckh and Clark, 2014). The simultaneous application of implantable optical microsensors in different parts and organs of an organism can provide researchers with an outstanding representation of the organism's physiological status at any given point in time. In the limiting case, the development of these techniques may allow the 3D-scanning of physiological parameters of the organism both *in vivo* and in real time to identify its stress state or initial stages of diseases.

One of the most promising types of implantable sensors is fluorescent molecular probes that are immobilized into microcapsules, or the so-called microencapsulated biomarkers (MBMs) (Sadovoy et al., 2012). The functional parts of these sensors are the dyes, whose spectra of fluorescence are sensitive to specific parameters of the media such as pH, ions, and some metabolites (Johnson and Spence, 2010). The dyes are encapsulated into semipermeable microcapsules to prevent their distribution in fluids of the organism, which allows their fluorescent signal to be enhanced and excludes from consideration a possible influence of dye or multi-sensing dye mixtures on the organism.

In a recent study, the applicability of MBMs for stress assessment has been shown on amphipods when MBMs were injected and visualized in the circulatory system of these crustaceans (Gurkov et al., 2016). Moreover, MBMs injected into the pericardium showed no influence on the development of *Danio rerio* (zebrafish) embryos for up to one week after the injection (Sadovoy et al., 2012), which is a sign of low or no toxicity of MBMs. In the present study, we applied MBMs in adult fish, and developed a technique for the parallel tracking of pH both in the bloodstream inside gill capillaries and in the interstitial fluid of muscles in adult zebrafish.

## RESULTS AND DISCUSSION

### Calibration of pH-sensitive MBMs

**Fig. 1.**
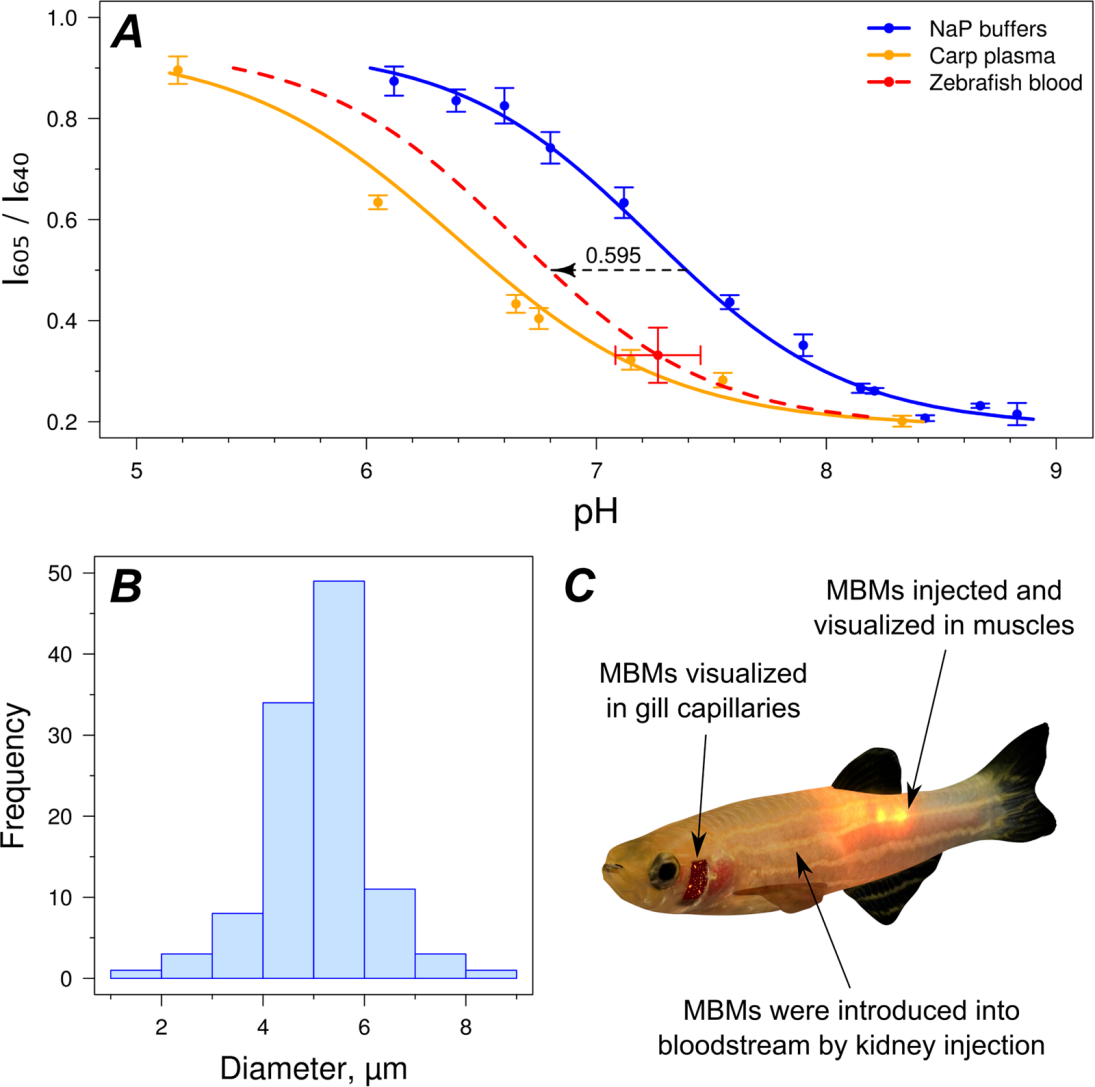
**Calibration of fluorescent microencapsulated biomarkers (MBMs), and visualisation of MBMs in fish for *in vivo* pH measurements.** (A) Calibration curves of the prepared pH-sensitive MBMs in sodium phosphate buffers, in *C. carpio* plasma, and in zebrafish blood. Putative calibration curve for MBMs in zebrafish blood was obtained by shifting the curve for buffers to fit the measurement in extracted zebrafish blood. For all measurements, the mean±s.d. is depicted. (B) Size distribution of prepared MBMs. (C) Scheme of zebrafish showing specific places where pH was measured with fluorescent MBMs.

MBMs containing pH-sensitive SNARF-1 were first calibrated in a number of buffers. However, we observed a significant mismatch between the obtained calibration curve and the measurements of pH and *I*_605_/*I*_640_ in zebrafish blood (Fig. 1A). SNARF-1 is known to have a shifted response to pH due to the influence of ions and biological molecules, which may be one explanation for the difference (Mölich and Heisler, 2005). Additionally, this mismatch can be explained by the Donnan effect, which was previously observed for microcapsules (Haložan et al., 2009; Sukhorukov et al., 1999).

Following both possible explanations and the effects observed in the previous studies, we can predict that the slope of the calibration curve would not be affected by the transfer of MBMs to media with different ion and protein composition and that there is only an additive shift for the whole curve. To confirm this hypothesis, we calibrated MBMs in blood plasma extracted from *Cyprinus* *carpio* (Fig. 1A). Indeed, the additive coefficients (Eqn 1) *b* of the obtained calibration curves differed dramatically (7.26 and 6.49 for buffers and plasma, respectively), but the slope coefficients *a* were similar (−0.394 and −0.462 for buffers and plasma, respectively).

The calibration curve obtained for the plasma of *C. carpio* did not show perfect agreement with the measurements obtained in zebrafish blood. To ensure the best quality of calibration of MBMs for use in zebrafish blood, we modified the calibration curve obtained in buffers and used it for further measurements. The additive coefficient *b* of the curve was decreased by the difference in pH between zebrafish blood and the buffer curve, which was ∼0.6 pH units (Fig. 1A). The obtained putative calibration curve was used for the pH measurements with MBMs in the gill capillaries and muscles of zebrafish.

The shape of the curve shows that in zebrafish blood, MBMs should be sensitive to pH in the range of ∼5.6–8.0. It is worth noting that the curve was not verified in the interstitial fluid of zebrafish muscles and that the measured absolute pH values in this case may have been slightly shifted; however, the values of the further investigated pH changes should not have been affected.

### Monitoring pH at normal state

MBMs with a median size of 5.2 μm (Fig. 1B) were introduced into the circulatory system of fishes through a kidney injection to distribute them throughout the body and to be visualized in the capillaries of the gills. Additionally, MBMs were injected directly into muscles (Fig. 1C). Immediately before the injections and all pH measurements, fish were anesthetized.

**Fig. 2.**
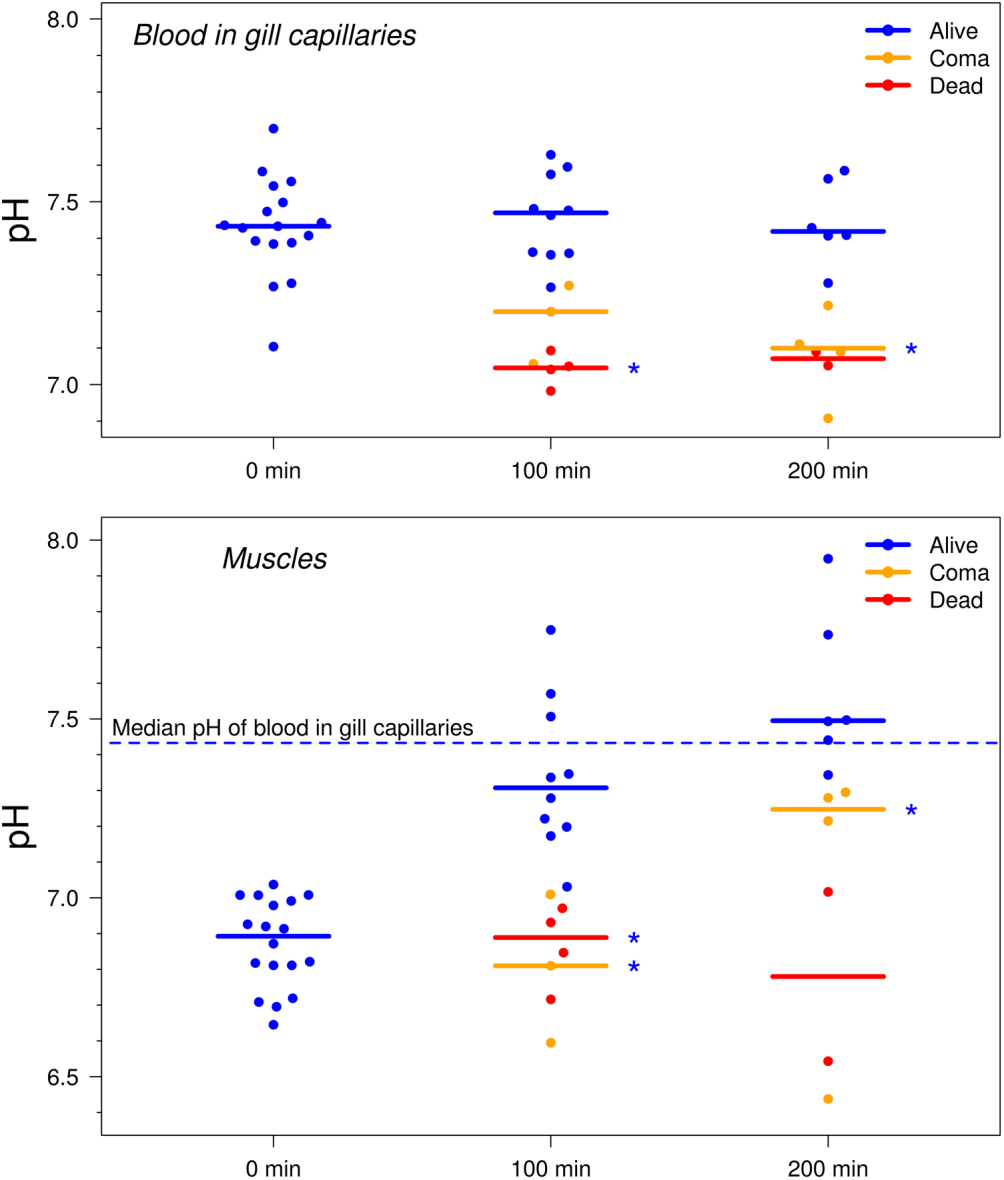
**pH measurements in gill capillaries and interstitial fluid of muscles of fishes immediately after the injection of MBMs and at 100 and 200 min after the injections.** Experiments were replicated three times; *n*=17 individuals; dead animals were excluded from the experiment after one measurement. Solid lines represent the median values. Dashed blue line shows the median pH of blood in gill capillaries of fish at normal state for all time points. *Indicates statistically significant difference from group of fish at normal state at the respective time point with *P*<0.05 (Dunn's test with Hommel's correction).

The median pH of blood measured with MBMs in gill capillaries at normal state (Fig. 2) remained stable in the range of 7.4–7.5 throughout the 200 min of the experiment (Kruskal–Wallis test *P*-value=0.69; Friedman test *P*-value=0.31). Unlike the blood pH, the pH observed in muscles of live fishes in the normal state showed statistically significant changes during the time of observation (Kruskal–Wallis test *P*-value <0.001; Friedman test *P*-value=0.011). The median pH measured immediately after the injection of MBMs into muscles was significantly acidic, at approximately 6.9. After 100 and 200 min of observation, the pH in muscles shifted closer to the median blood pH and was significantly different from the initial measurement (both *P*-values <0.001). The median pH values identified at 100 and 200 min after injection were approximately 7.3 and 7.5, and were not significantly different (*P*-value=0.25).

The observed stability of the blood pH in gills at a normal state indicates the good repeatability of the MBMs' readout and that injecting the MBMs did not influence the general blood pH. Although a normal blood pH in the range of 7.7–8.0 has been demonstrated for most fish species (Evans and Claiborne, 1997; Tzaneva et al., 2011), a pH range of 7.3–7.6 has been reported for some species (Imsland et al., 2008; Mandelman and Skomal, 2009; Skomal, 2007), which is in accordance with our measurements. However, it must also be considered that the applied anesthesia could have led to somewhat decreased pH values during the optical measurements (Iwama et al., 1989).

For injections, we dissolved MBMs in unbuffered normal saline solution with pH ∼5.9. Although only a small amount of the solution was injected, it might have caused the observed acidic pH in muscles immediately after the injection. Additionally, we cannot neglect the possible input of cells opening to the identified acidification because cytosol and, in particular, some cell compartments have relatively decreased pH values. During the rest of the experiment, the interstitial pH at the injection site returned to values similar to the measured blood pH.

### pH dynamics in comatose and dead fishes

In addition to tracking the pH in fishes under normal physiological conditions, we monitored the pH in comatose and dying individuals. Repeated anesthesia can be associated with intoxication, after which some fish cannot be awakened. The percentage of fish that fell into a coma is probably dependent on the natural variability in the health status of the subjects. The state is caused by oxygen deprivation in the brain, which generally results from poor gill ventilation and decreased heart rate. We applied the chosen anesthesia procedure intentionally to induce significant disturbances in metabolism and show the functionality of pH-sensitive MBMs. The pH values in dead fish were measured just once after death and were excluded from the experiment after the 100 min time point.

The median blood pH in the gills of a comatose fish (7.20) was lower than that in fish at normal state (7.47) when measured 100 min after the injection, but the difference was not statistically significant (*P*-value=0.072). Dead individuals (median pH 7.05) were significantly different from the group at normal state (*P*-value=0.019). At 200 min after the injection, only two comatose fish had died, and their median blood pH (7.07) was considerably lower but was not significantly different from the respective measured pH (7.42) for alive fish (*P*-value=0.18). Fish in a coma at the 200 min time point showed a median blood pH similar to that of dead fish (7.10), but their number was higher, and the difference between them and the control group was statistically significant (*P*-value=0.042).

In the case of muscles, the pattern was similar. Median pH in muscles of both comatose and dead individuals (6.81 and 6.89) at 100 min after the injections was significantly more acidic (*P*-values=0.045 and 0.019) than the corresponding measurements in live fish (7.31) and was even slightly lower than that observed immediately after the injection (6.89). At 200 min after the injection, three fish had entered a coma; the median pH for the four comatose animals (7.25) was significantly different from the pH in muscles (7.50) of fish at normal state (*P*-value=0.042). Two dead individuals also showed low median pH values in muscles (6.78), but these values were not significantly different from the control group (*P*-value=0.18).

The observed acidification decreased the blood pH and prevented pH in muscles from recovering, which is probably related to hypoxia because comas are associated with poor gill ventilation. Hypoxia induces the production of acidic metabolites such as lactate, which is widely known to cause the acidification of an organism's fluids (Philp et al., 2005). Importantly, muscles seem to be more sensitive to hypoxia than blood in gills as a reaction of muscles for fish in a coma was identified earlier. This effect is expected because gills are the main organ to import oxygen and excrete carbon dioxide for acid-base regulation. However this effect can also be enhanced by active restoring muscle pH in normal state, which may also increase the differences with the comatose group, where the resources for pH regulation are limited. Therefore, the blood pH in gills and pH of interstitial fluid in muscles can be considered markers that are applicable for different purposes: the first describes general response of the whole organism, whereas the second seems to be more sensitive to starting physiological changes.

### Comparison of readouts of MBMs and microelectrode

**Fig. 3.**
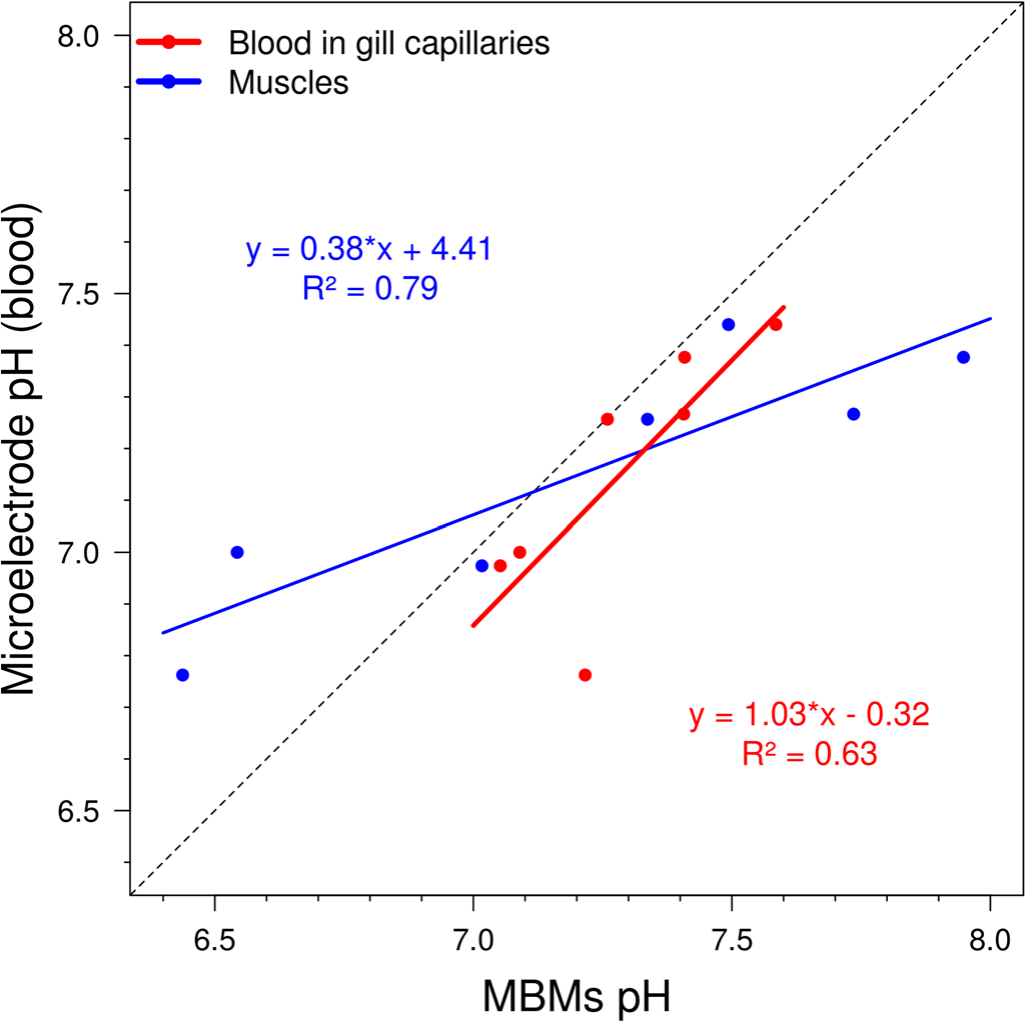
**Comparison of pH values measured by MBMs in gill capillaries and muscles with pH measurements in extracted blood measured using a microelectrode.** Performed 200 min after injection for part of animals in different physiological states; *n*=7 individuals.

Immediately after the experiment, we extracted blood from some live, comatose, and dead individuals and measured their blood pH with a microelectrode. These data are well correlated with the values obtained by MBMs both in muscles and in gill capillaries (Fig. 3). Moreover, the regression line between the blood pH measurements by MBMs and microelectrode is almost perfectly parallel to the hypothetical line of ideal concordance between the measurements, but the readout of the microelectrode is more acidic than the signal of MBMs by approximately 0.13 pH units. This shift is similar to the difference of 0.17 between the blood pH measured by the microelectrode for calibration of MBMs and the median blood pH measured in gill capillaries throughout the experiment. These differences probably reflect the acidification of blood due to an interaction with air after the extraction and show the advantage of MBMs for *in loco* pH measurements directly in the bloodstream.

### MBMs as a promising tool for *in vivo* pH monitoring in fishes

Overall, the obtained results demonstrate the applicability of MBMs as pH sensors for repeated *in vivo* measurements in both the bloodstream of gills and interstitial fluid of muscles in parallel. It must be noted that the described procedures for pH measurement may have resulted in slightly altered absolute values of pH, although the observed pH changes should be precise. Fish anesthesia is known to cause blood acidification, especially at a stage with cessation of opercular movements (Iwama et al., 1989) that is required for the rapid measurement of blood pH when using the suggested optical technique. Moreover, in this study, we could not calibrate MBMs directly in the interstitial fluid of muscles. Thus, for measurements in muscles, we had to rely on a calibration curve for blood, which could also have led to slightly altered absolute pH values. For more prolonged experiments in the future, there may be a need for another method of fish immobilization (at least, a more delicate anesthetic technique) or in the fixation of a flexible optical fiber to an organ of interest (for pH measurements without any physiological disturbances) and a more precise method for injecting MBMs into the bloodstream. Nevertheless, the proposed technique offers a new level of quality for *in vivo* pH screening in fish.

## MATERIALS AND METHODS

### Preparation of pH-sensitive MBMs

pH-sensitive MBMs were prepared as previously described (Gurkov et al., 2016). The fluorescent dye SNARF-1 conjugated with dextran (SNARF-1-D; Thermo Fisher Scientific, D-3304) was co-precipitated in porous microcores of CaCO_3_ by mixing 2 ml of 2.5 mg ml^−1^ SNARF-1-D solution with 0.615 ml of 1 M CaCl_2_ and 1 M Na_2_CO_3_ solutions. The cores were then covered by 12 layers of oppositely charged polyelectrolytes poly(allylamine hydrochloride) (PAH; Sigma #2832315) and poly(sodium 4-styrenesulfonate) (PSS; Sigma #243051) and by the final layer of poly(L-lysine)-graft-poly(ethylene glycol) co-polymer (PLL-g-PEG; SuSoS, SZ34-67). The outmost layer of PLL-g-PLL increased the biocompatibility of the formed microcapsules (Sadovoy et al., 2012). After dissolving CaCO_3_ cores in 0.1 M EDTA solution (pH 7.1), the MBMs had the following structure: SNARF-1-D/(PAH/PSS)_6_/PLL-g-PEG.

The concentration and size distribution of prepared MBMs were determined using a Mikmed-2 fluorescent microscope (LOMO, Russia) with a hemocytometer followed by analysis in the Fiji software (Schindelin et al., 2012, 2015).

### pH measurements and calibration of MBMs

SNARF-1 is sensitive to pH values in range of 6-9 (Johnson and Spence, 2010). The fluorescent dye has two spectral peaks, and the ratio between the fluorescence intensities at two wavelengths from these spectral regions can be used for direct pH measurements. In this study, we calibrated the microencapsulated SNARF-1-D and further identified the pH *in vivo* by calculating the ratio of fluorescence intensity at 605 nm to 640 nm (*I*_605_/*I*_640_; Gurkov et al., 2016). MBMs were visualized (both during calibration and *in vivo*) in the red channel using a Mikmed-2 fluorescent microscope (using 40× objective lens) coupled with a QE Pro spectrometer (OceanOptics).

pH-sensitive MBMs were calibrated by dissolving in a series of sodium phosphate buffers (in pH range 6.1–8.8), in the blood plasma of *C. carpio* (in pH range 5.2–8.3), and in blood from *Danio rerio* (zebrafish; at one pH point; *n*=6). Measurements of *I*_605_/*I*_640_ value in buffers and plasma were obtained 10-15 times at each pH point. Blood from *C. carpio* was obtained from the caudal vein of anesthetized fish using a heparinized syringe. Plasma was separated by centrifugation at 2000 ***g*** for 5 min. Whole blood was obtained from anesthetized zebrafish after tail cut by centrifugation of fish at 40 ***g*** for 1 min (Babaei et al., 2013). Reference measurements of pH in blood (*n*=6; measured separately from *I*_605_/*I*_640_) and plasma were performed with an InLab Nano microelectrode (Mettler Toledo).

The relation between pH and average readout of MBMs (I_605_/I_640_) was fitted by nonlinear regression to a model with two variables (Eqn 1) (Bottenus et al., 2009). The coefficients 0.19 and 0.94 are equal to the minimal and maximal observed values of *I*_605_/*I*_640_, respectively.
(1)
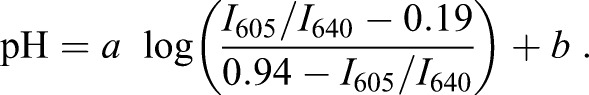


### Experimental procedures

All experiments were carried out on adult *D. rerio* (line with impaired pigmentation) weighing 0.26±0.08 g (in accordance with EU Directive 2010/63/EU for animal experiments). Before injection and each pH measurement, fish were anesthetized by immersion into 0.1 µl ml^−1^ suspension of clove oil (Eugenol) in water for 1.5 min (Cheung et al., 2014). For MBM implantation, individuals were immobilized on a wet sponge, and MBMs suspension in normal saline solution (∼6×10^5^ microcapsules µl^−1^) was injected using a 31 G needle (Ø 0.25 mm) connected to a microinjector IM-9B (Narishige, Japan). For pH measurements in blood of gill capillaries, 2 µl of the MBM suspension were injected into a central bulge of the trunk kidney, which is known to be enriched by a net of capillaries. Fish were rinsed by water, and one operculum was resected by scissors to easily visualize MBMs in gill capillaries. Intramuscular injections of 1 µl of MBM suspension was made into the dorsal portion of the tail, caudal from the dorsal fin. During signal acquisition from the MBMs inside the gill capillaries, the gills were moistened with water to keep them wet. After signal recording, fish were returned to the water tanks with aeration for recovery (normally takes ∼5 min).

A comatose state was diagnosed when the fish did not become rebalanced for more than 30 min, had negligible opercular movement, and did not respond to fin pinch but the circulation of blood cells in the capillaries of the fins could clearly be seen under a microscope. Death of the animal was diagnosed by the absence of any blood flow in the capillaries of fins.

The experiment was repeated three times, and each replicate included at least five animals. Statistically significant differences between experimental groups were investigated by a Kruskal–Wallis (in case of repeated measurements Friedman test was also applied) followed by a Dunn's test with Hommel's correction for multiple comparisons in the statistical environment R (www.R-project.org/) using *PMCMR* (CRAN.R-project.org/package=PMCMR). Fig. 2 was plotted using *beeswarm* (CRAN.R-project.org/package=beeswarm).
